# Digital inequalities and U.S. undergraduate outcomes over the first two years of the COVID-19 pandemic

**DOI:** 10.1371/journal.pone.0319000

**Published:** 2025-03-03

**Authors:** Vikki S. Katz, Amy B. Jordan, Katherine Ognyanova

**Affiliations:** 1 School of Communication, Chapman University, Orange, California, United States of America; 2 School of Communication & Information, Rutgers University, New Brunswick, New Jersey, United States of America; Marquette University College of Education, UNITED STATES OF AMERICA

## Abstract

The COVID-19 pandemic necessitated major and rapid changes to undergraduate student learning conditions, but the longer-term consequences of those changes have yet to be fully understood. We assess how *digital inequality*—defined as challenges in accessing or maintaining a broadband internet connection and functional digital devices—affected undergraduates’ advancement toward on-time graduation over the first two years of the pandemic (2020-2022). We utilize survey data from a representative sample of 1,106 undergraduates at Rutgers University-New Brunswick, a public, 4-year university located in New Jersey, USA. Respondents were undergraduates who had been enrolled in full-time student status throughout the first two years of the pandemic. We examine how digital inequality and other sociodemographic factors affected their persistence in full-time student status toward on-time graduation using structural equation modeling. Results show that students with inadequate or inconsistent internet and digital devices developed less remote learning proficiency than their better-connected peers. They were more likely to report having taken incomplete grades in specific courses and/or to have extended their time to graduation. We discuss the implications of these findings for developing digital components to campus emergency planning and for efforts to ensure more equitable learning experiences for undergraduate students in non-emergency periods in the aftermath of the pandemic.

## Introduction

The sudden onset of the COVID-19 pandemic created or exacerbated a broad swath of challenges for millions of U.S. undergraduate students. The hasty retreat from campus to complete their Spring 2020 coursework online was enormously disruptive for all students. It was especially challenging, however, for students who were “under-connected,” meaning that their internet access and/or digital devices could not consistently or adequately support their remote learning needs [1].

In April and May 2020, the authors gathered cross-sectional data from more than 3,000 U.S. undergraduates nationwide to capture their experiences in those first weeks of remote learning. Our results revealed that under-connected students struggled most in the sudden pivot to remote instruction, and we identified key factors and sociodemographic differences that contributed to those challenges [2].

In this article, we build on those 2020 findings by investigating how students fared in the longer-term phase of remote (and then hybrid) learning that began in Fall 2020, when instructors had had more time to prepare to teach via online modalities and students had become more acclimated to online learning environments. Drawing on additional survey data we collected in Spring 2022, we ask: Did digital inequality affect undergraduates’ advancement toward on-time graduation over the first two years of the COVID-19 pandemic?

### Undergraduate trajectories in the U.S. amidst the COVID-19 pandemic

The COVID-19 pandemic had immediate and substantial effects on U.S. students’ matriculation and persistence in higher education. Students who had planned to matriculate into higher education in Fall 2020 often did not do so. Liu sheds light on whose postsecondary plans were most likely to be upended by the pandemic, using U.S. Census Bureau Household Pulse Survey data from August 2020 through March 2021 [3]. She finds that almost three-quarters of households reported disruption in educational plans during that period, but that Black and Latino respondents were more likely to indicate that they had canceled their plans, whereas White respondents were more likely to report taking coursework in alternate formats [3].

Among the 2.6 million students who began college in Fall 2019, one in four (26%) did not return to continue their studies in Fall 2020 [4]. At public four-year universities (like Rutgers University-New Brunswick, where this study was conducted), there was a 2% drop in the number of enrolled students pursuing a bachelor’s degree between Spring 2020 and 2021, and another 3.5% drop between Spring 2021 and 2022 [5]. These data also reveal that students who stopped out or dropped out of college during the first two years of the pandemic were more likely to be Hispanic or Black than to be White or Asian [4]. Furthermore, a number of studies, including international ones, indicate that re-enrollment after taking a leave of absence was less likely during the acute phase of the pandemic than in other time periods [6].

And among students who completed their undergraduate degrees shortly before or during the acute phase of the pandemic, evidence indicates limited success entering the workforce. The 2021 National Survey of College Graduates revealed large increases in unemployment and part-time employment (as opposed to full-time employment) for U.S. undergraduates who had completed their college degrees between 2019 and 2021 [7]. Those who were unemployed or employed part-time indicated the pandemic was a major factor in why a full-time job was unavailable to them or why their hours had been reduced. Analyses also revealed that Hispanic and Black college graduates were more likely to be experiencing pandemic-related adverse employment circumstances than their White counterparts [7]. Furthermore, recent U.S. college graduates were more likely to be unemployed than any other adult age group in 2022 and 2023 [8].

Taken together, these pandemic-related shocks at every stage of students’ postsecondary educational trajectories suggest particularly worrisome consequences for a generation of progress with regard to improving access to higher education within the U.S., and particularly for students from historically marginalized backgrounds [9–11].

Studies of undergraduate experiences during the onset, and then acute phases, of the pandemic frequently highlight that students’ vulnerabilities to shocks to their educational trajectories were determined by how their challenges compounded over time. Some researchers operationalize students’ vulnerabilities across economic, social, mental health, and learning domains [3]; others measure their cumulative stressors [12]. In a variety of ways, researchers have sought to identify and measure the structural disadvantages that made some students more susceptible to compounded disadvantages that could compel them to stop out, or drop out, of college over time.

Given that digital access—to high-quality broadband internet, and to a computer in good working order—were so central to students’ abilities to be fully engaged in their courses during the pandemic, we see students’ digital challenges as having been under-theorized and imprecisely measured in many studies conducted during this unique time period. Below, we argue that a precise conceptualization of students’ experiences of digital inequality is necessary to fully account for the vulnerabilities and stressors that students faced over two years of remote and hybrid learning following the onset of COVID-19.

### Digital inequality and U.S. undergraduates’ pandemic learning experiences

Digital inequality disproportionately affected lower-income, first-generation, and racial/ethnic minority college students in the U.S. even before the pandemic [13,14]. While U.S. university campuses offer students free WiFi access and have computer labs and loaner laptops available, broadband internet connectivity remains high cost in many communities. By 2022, average U.S. household spending on home broadband access had reached $74 per month [15], a prohibitive cost for low-income households that nonetheless does not account for families having to upgrade services during lockdown, when multiple family members were likely to be streaming school- and work-related content simultaneously.

Research conducted in the U.S., Italy, and Switzerland in April 2020 revealed that U.S. households were also less well-resourced at the start of the pandemic lockdown than their European counterparts. Ten percent less Americans owned a computer as compared with Swiss or Italian respondents, and Americans were more than twice as likely to only have one internet-connecting device at home [16]. Accessible and affordable options for repairing or replacing hardware are likewise challenging to find in the U.S. Even pre-pandemic, U.S. students often struggled with the “dependable instability” of their devices [13] and had to rely on WiFi in libraries, fast food restaurants, and other community locations when not on campus, belying the ways in which this population has often been “presumed connected” [17].

As the above suggests, the digital challenges experienced by college-aged U.S. Americans are not a complete, static absence of internet access or internet-connecting devices. Rather, they face challenges by virtue of being *under-connected*, meaning that their internet connectivity and/or their digital devices are too inadequate or inconsistently functional to meet their needs [1]. When we surveyed more than 3,000 students in April and May 2020, 42% of our sample reported that, in the year prior to remote learning, they had been under-connected in at least one of three key ways: their internet had been cut off due to inability to pay, they had hit the cap on their smartphone’s data plan before the end of a month, and/or they had had a broken laptop for 10+ days (not necessarily consecutively) [18]. Experiencing digital access as unreliable and unpredictable is a stressor that compounds other challenges that undergraduates face as they pursue higher education.

Furthermore, under-connected students are more likely to come from lower-income families and to report personal or family financial instability because maintaining broadband connectivity and purchasing digital devices is expensive [2,14]. Under-connected college students are also more likely to be members of underrepresented minority groups and/or first-generation college goers [13]. Thus, there is a meaningful convergence between the undergraduate students most likely to have stopped out or dropped out during the first two years of the COVID-19 pandemic, as summarized in the prior discussion, and those most likely to report being under-connected during remote and hybrid learning.

Some prior studies have acknowledged digital inequality as part of their efforts to understand U.S. students’ pandemic learning experiences. For example, a cross-sectional survey of 162 students conducted in April 2020 at William Paterson University, another public university in New Jersey, found that 59% of students reported difficulties with online learning modes and 22% specifically referenced inadequate WiFi or computer access [19]. A survey of 1,220 students at Georgia State University examined students’ stressors in April and May 2020, finding that access to technology and WiFi was a “strong” or “extreme” stressor for 25% of their sample, and a “moderate” stressor for another 28% [20]. While both studies contribute to our understanding of students’ pandemic experiences by acknowledging digital challenges, the authors conflate what digital inequality scholars have long treated as distinct digital challenges by asking about internet access and device access in a single, double-barreled question.

In a class-level study, Gillis and Krull surveyed their introductory sociology courses at three time points in March and April 2020, mainly to assess students’ responses to their different approaches to adapting the same material for emergency remote instruction [21]. Interestingly, they found that 58% of students who anticipated that they would have no issues with internet connectivity that affected their course participation did, in fact, experience such interruptions, whereas students who anticipated such interruptions were generally correct in those predictions [21]. This study is consistent with pre-pandemic research on under-connected students’ awareness of their dependable digital instability [13], and with our own conceptualization of how under-connectedness functions within students’ day-to-day stressors on their learning [2].

A study by Digital Promise more closely mirrors our own approach by distinguishing between digital challenges stemming from internet connectivity and devices. Their survey of 717 four-year university students in May 2020 found that 44% had connectivity challenges that impacted their abilities to participate fully in their courses [22]. Sixteen percent reported problems with digital devices that interfered with their ability to complete their coursework. They also note significant differences in students’ challenges by race/ethnicity and by household income [22].

In our own prior study, we conducted an anonymous online survey of 3,113 undergraduates at 4-year U.S. universities and colleges in 19 states and the District of Columbia in April and May 2020 [2], with the goal of capturing students’ experiences in emergency remote learning as they were unfolding. In that survey, we develop what is, to our knowledge, the most systematic assessment of digital inequality among U.S. college students during the pandemic. Our measures begin by capturing what types of internet access and digital devices they have regular access to for remote learning. We included previously validated under-connectedness measures that we had developed for research with K-12 populations [1,23] to identify which students came into remote learning with a recent history of digital challenges, in the forms of interrupted home internet access, having hit data plan caps, or having an inoperable computer for 10 or more days at least once in the prior year. Against that backdrop, we adapted previously validated measures of under-connectedness to capture the experiences they were having during remote learning. We measured connectivity challenges by asking respondents whether their internet connection was slow; they could reliably livestream coursework; had reliable access to pre-recorded lectures; and the ability to download large files [2]. We measured device challenges by whether they were relying on devices that were in poor working condition to complete remote schoolwork; had to rely on a smartphone to complete schoolwork; and/or had to share the device they relied on for remote learning with other people [2].

In a 2021 article from these data, published in *PLoS ONE*, we present a structural equation model that tests how being under-connected in the prior year, device challenges, connectivity challenges, and challenges communicating with instructors in emergency remote learning (in addition to a broad set of socio-demographic control variables) explain variations in which students quickly developed a sense of *remote learning proficiency.* We operationally define remote learning proficiency as students’ self-reported confidence that they: (a) understood what instructors were expecting of them; (b) could keep track of deadlines and due dates; and (c) felt they could effectively use the digital platforms and programs required for remote coursework [2].

We found that students who reported connectivity and device challenges were significantly less likely to report remote learning proficiency than students who were not under-connected. Furthermore, because communicating with instructors required digital connectivity (through, for example, Zoom office hours), we found that challenges communicating with instructors were also significantly associated with lower remote learning proficiency [2]. Prior to the pandemic, lower-income and first-generation students were less likely to reach out and let instructors know when they were struggling in their coursework [24–26]; our findings suggested those patterns were replicated in remote learning conditions as well.

Our 2020 study findings offered insights into the effects of digital inequality on undergraduates’ persistence in completing their Spring 2020 courses, and how students’ remote learning proficiency related to variations in students’ socio-demographic characteristics and early pandemic learning experiences [2]. In the current article, we build on the insights of the prior study to examine longer-term effects of connectivity, device, and communication challenges on students’ remote learning proficiency. Thus, we begin with hypotheses to test whether the model we developed with our 2020 data also fits the 2022 data:

**H1:** Students who reported more connectivity challenges since the onset of the pandemic will feel less proficient as remote learners, compared with students who report fewer connectivity challenges.

**H2:** Students who reported more device challenges since the onset of the pandemic will feel less proficient as remote learners, compared with students who report fewer device challenges.

**H3:** Students who reported more challenges communicating with their instructors since the onset of the pandemic will report feeling less proficient as remote learners, compared with students who report fewer communication challenges.

**Fig 1:**
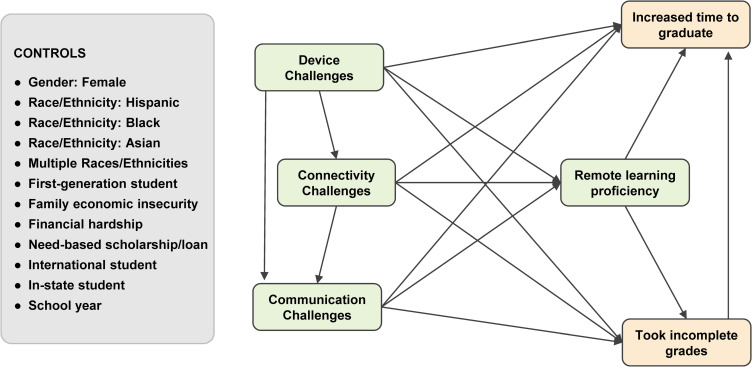
Hypothesized structural model.

We then move beyond the focus of the 2020 study to investigate how those factors relate to undergraduate students’ progress toward degree completion, measured by on-time completion of individual classes and progress toward on-time graduation, as expressed in our hypothesized model (see Fig 1). Our goal in doing so is to examine how inequalities in digital access, over time, may not only have constrained students’ sense of digital competence (i.e., remote learning proficiency), but also, their concrete educational outcomes as a result of those inequalities in digital access and skills.

### Digital inequality and U.S. undergraduate learning outcomes

From the onset of the COVID-19 pandemic, students were concerned about how remote learning would constrain their abilities to complete their undergraduate degrees. Aucejo and colleagues surveyed 1,500 students at Arizona State University, another diverse, public U.S. university, in November 2020 [27]. Even at that early stage of the pandemic experience, 13% of students reported delaying graduation, and lower-income students were 55% more likely to have done so than their higher-income peers. The number of students who expected to take a break from their undergraduate degrees in Fall 2020 was twice the historical rate, and 11% reported that they had withdrawn from a course in the Spring 2020 semester [27]. While their data unfortunately do not account for digital inequalities within their sample, the authors do find that lower-income, non-White, and first-generation students were more likely, even six months into the pandemic, to have delayed their expected graduation term. This suggests digital inequality is a confounding factor in their analysis, given that race/ethnicity, income, and parental education all closely correlate with being under-connected.

As Velez points out, uncertainty was a hallmark of the acute pandemic period, disrupting not only normative progress through coursework and toward degree completion, but also students’ sense of self and the plans they had for themselves [28]. Students found remote coursework more difficult, but also less rigorous [22]. They spent a lot of time managing being under-connected. We found that between one-quarter and one-third of surveyed students at University of Virginia and at Rutgers University in 2022 reported having to complete schoolwork in a library, coffee shop, or restaurant because their home internet connection was not reliable or fast enough; staying up later to do schoolwork because their home internet was faster at night; or, having to ask people they live with to get offline to they could live stream classes either “sometimes” or “often” during remote or hybrid learning [18].

Students also struggled with time management, self-motivation, and managing responsibilities as workers and caregivers [29,30]. In interviews conducted with undergraduates at Rutgers University who had been enrolled full-time for the first two years of the pandemic, Laliberte found that students felt they were working harder for less reward. Students reported that it was difficult to connect with peers and feel a sense of belonging to classes and to student life, and more difficult to master challenging coursework with the many distractions of living at home [31].

Based on these insights into how challenges evolved for students over time, we extend our 2020 model by examining how digital inequalities and remote learning proficiency relate to students’ likelihood of having delayed completion of course-level work (in the form of incomplete grades) and delayed time to graduation, as a more global measure of slower progress to degree:

**H4:** Students who report lower remote learning proficiency will be more likely to report taking incomplete course grades than students who report higher remote learning proficiency.

**H5:** Students who report lower remote learning proficiency will be more likely to report extending their time to graduation than students who report higher remote learning proficiency.

## Materials and methods

### The current study

In February and March 2022, we surveyed 1,106 undergraduates who had been enrolled full-time at Rutgers University-New Brunswick since the onset of the COVID-19 pandemic, in Spring 2020. The data were collected through an anonymous, 10-minute online questionnaire using the Qualtrics platform. The study was approved by the Rutgers University Institutional Review Board.

This cross-sectional survey captured Rutgers undergraduates’ perspectives on how their experiences in fully remote (Spring 2020 through Summer 2021) and then hybrid (Fall 2021 through Spring 2022) learning environments had evolved over the prior two years. Because the survey focused on students who had been enrolled full-time at Rutgers throughout the first two years of the pandemic, our dataset intentionally reflects a “best-case” student population, in order to isolate the effects of digital inequality on pandemic learning even among students who managed to persist in full-time status. The sample parameters enable more consistent comparisons of students’ experiences—and of how they evolved over the first two years of the pandemic—than would have been the case had we also included students who switched to part-time status or who had temporarily or permanently discontinued their studies, since those students have had materially different experiences of pandemic learning.

### The study site

Our data provide representative insights into the experiences of students who managed to persist in full-time enrollment status from Spring 2020 to Spring 2022 at Rutgers University-New Brunswick (see Table 1).

**Table 1:** Unweighted demographics of survey respondents and full sample demographics.

Variable	Percent in the study sample	Percent in the study population
Female	66	50
Male	34	50
Race/Ethnicity: Asian	41	42
Race/Ethnicity: Black	8	6
Race/Ethnicity: Hispanic	11	9
Race/Ethnicity: White	46	34
Multiple Races/Ethnicities	17	NA*
First-generation students	29	NA*
Living on campus	11	NA*
In-state students	87	85
Out-of-state students	6	6
International students	7	9

*University-level data for these subgroups were not available to the researchers.

The State University of New Jersey, Rutgers is the oldest, largest, and top-ranked public university in the New York/New Jersey metropolitan area. It is also one of the most racially, ethnically, and economically diverse universities in the U.S. Rutgers has campuses in three New Jersey cities, and approximately 82% of students are New Jersey residents. The largest campus (with 43,000 enrolled students) is located in New Brunswick, the site of data collection for this study. By focusing on an undergraduate student population that is unusually diverse, we were able to parse the effects of digital inequality on on-time progress toward graduation in our analyses, in the context of socio-demographic differences related to racial/ethnic identity, gender identity, first-generation status, financial instability, and other factors that prior research confirms have been important drivers of variance in pandemic educational experiences.

On March 10, 2020, Rutgers announced that classes would be cancelled through the end of spring break and that instruction would be remote for at least the first two weeks after spring break. On March 17, 2020, the university president announced that all in-person classes, assessments, and events would be suspended through the end of the semester. The 2020 commencement activities were held online. On July 6, 2020, the incoming university president announced plans for most classes to be delivered remotely, with in-person instruction for Fall 2020 limited to critical face-to-face coursework (e.g., medical specialties and scientific laboratory work). Instruction, assessments, and exams remained remote through the 2020-2021 academic year.

On March 25, 2021, Rutgers announced it would require the COVID-19 vaccine for students enrolled for the fall 2021 semester. The 2021-2022 school year was a mix of hybrid formats, with some coursework still being taught entirely remote and many classes proceeding with a mix of in-person and online instruction, assessments, and exams. By limiting the study sample to students who had been enrolled full-time through emergency lockdown (in Spring 2020), a fully remote academic year (2020-2021), and a hybrid format year (2021-2022), our analyses capture the experiences of a diverse undergraduate population through three distinct phases of pandemic learning, requiring varying levels of digital connectivity for full academic engagement.

### Sample demographics

Table 1 reflects the demographics of the study sample, who reported ages ranging from 19 to 40 (*M*=21.2, *SD*=1.4), with 94% of respondents between ages 19 and 22. The participants were 66% female and 34% male. A total of 8% reported identifying as Black, 41% as Asian, 11% as Hispanic, and 46% as White. The numbers sum to over 100% because respondents could select more than one racial or ethnic identity (17% of respondents did so). In addition, 7% of participants were international students. The ethnic and racial composition of the sample was similar to that of all students at Rutgers University-New Brunswick university who met our study criteria. Chi-squared tests comparing sample and population found significant differences for gender, percent Black students, and percent international students. To improve the representativeness of the sample, weights were generated and applied adjusting for gender, race, and being an in-state student. The next section reports weighted descriptive statistics, while unweighted ones are presented in Table 1.

### Measures

*Increased time to graduate* was recorded as a binary variable. Respondents were asked if they had had to reduce their course load, thereby taking more time to graduate during the pandemic. A total of 25% of the sample said “yes.”

*Taking incomplete grades* was also measured as a binary variable. Respondents were asked if they had had to take incomplete grades to remain in college during the pandemic; 33% selected “yes.”

*Connectivity challenges* were measured by asking participants how much they agreed that four statements reflected their general experience since the onset of the COVID-19 pandemic. Using a Likert-type scale ranging from 1 (*Strongly disagree*) to 5 (*Strongly agree*), the statements included (1) having a slow internet connection; (2) not being able to reliably livestream video; (3) having no reliable access to recorded lectures; and (4) not being able to download large files. The scale had good internal consistency (α =.83). The final measure (range 1-5, *M* = 2.5, *SD* = 1.1) was calculated by averaging the four items.

*Device challenges* were evaluated by asking respondents how much they agreed that three statements reflected their general experience since the onset of the pandemic. Using a Likert-type scale from 1 (*Strongly disagree*) to 5 (*Strongly agree*), the statements included (1) having to share devices with too many people; (2) using devices that were slow or in poor working condition; and (3) having to mostly use a smartphone to complete their schoolwork. The scale had satisfactory internal consistency (α =.77). The final measure (range 1-5, *M* = 2.1, *SD* = 1.0) was calculated by averaging the three items.

*Communication challenges* were measured by asking how much students agreed with four interrelated statements about their abilities to communicate with their instructors since the pandemic began. Using a Likert-type scale ranging from 1 (*Strongly disagree*) to 5 (*Strongly agree*), the items asked students about (1) not being to communicate with professors as much as they would like; (2) not being able to communicate with teaching assistants as much as they would like; (3) finding it easier to connect with or relate to professors; and (4) finding it easier to connect with or relate to teaching assistants. The last two items were reverse-coded to create a measure where a higher score meant having more challenges in connecting with instructors. The scale had acceptable internal consistency (α =.79). The final measure (range=1-5, *M* = 3.6, *SD* = 1.0) was calculated by averaging all items.

*Remote learning proficiency* was measured by asking participants how much they agreed with three statements on a Likert-type scale ranging from 1 (*Strongly disagree*) to 5 (*Strongly agree*). We asked students to agree or disagree with whether, in remote learning environments, they felt confident (1) understanding what instructors expected of them; (2) keeping track of deadlines and due dates; and (3) figuring out how to use programs they needed for their coursework (e.g., Zoom, Google Classroom). The items were reverse-coded so that a higher score meant having fewer issues navigating in fully or hybrid remote learning environments. The scale had satisfactory internal consistency (α =.73). The final *remote learning proficiency* measure (range 1-5, *M* = 3.0, *SD* = 1.2) was calculated by averaging the reverse-coded items, consistent with original development of the measure in analyses of the 2020 dataset.

*Control variables* in the model included gender identity, racial/ethnic identity, and year in college. We also included variables indicating whether participants were international (7%), in-state (87%), or out-of-state (6%) students. A control was also included for first-generation college students (41%), identified as those who reported that neither parent had a college degree.

Three variables were included to account for the financial situations of both respondents and their families. *Financial hardship* was measured by asking students whether their personal financial situation had taken a turn for the worse since the start of the pandemic. Responses were measured on a scale ranging from 1 (*Strongly disagree*) to 5 (*Strongly agree*) with *M* = 3.3 and *SD* = 1.4. *Family* e*conomic insecurity* was a dichotomous variable, coded 1 for participants who reported having families they felt were financially insecure (35%) and 0 for those who did not (65%). *Need-based scholarship or loan* was also dichotomous, coded 1 for participants who had received a need-based scholarship or taken out a student loan (72%) and 0 for those who did not (28%).

**Fig 2:**
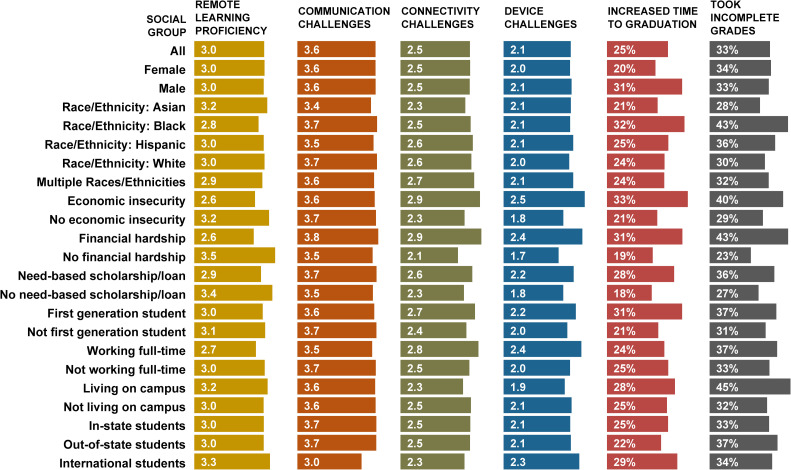
Weighted mean levels of model variables for key demographic groups.

Weighted mean levels of model variables by key demographic groups are presented in Fig 2. To put those variables in context, we further examined how levels presented here compare to those recorded in a previous cross-sectional sample of Rutgers undergraduates collected in 2020 by the authors [3]. While that data included students at multiple institutions, we extracted a smaller subset of respondents who were in their first or second year at Rutgers University in 2020 (*N* = 794). That corresponds to the sample examined here: students who were in their third year and above in 2022. Weighted t-tests comparing the two samples showed a significant increase in *remote learning proficiency* over time from 2.6 to 3.0, *t*(1881) = 4.6, *p* <.001, as well as a smaller increase in reported *communication challenges* from 3.4 to 3.6, *t*(1876) = 3.1, *p* <.001. The differences in means for *device challenges* and *connectivity challenges* were not significant. *Increased time to graduate* and *taking incomplete grades* were measured retrospectively and only in 2022. Since no identifiable information was collected from 2020 survey participants, the two samples could not be matched and are thus treated as repeated, cross-sectional data. Detailed information on variable levels across demographic groups is available in Tables A1 and A2 of Appendix A.

**Table A1:** Weighed mean outcomes in 2020 *(N*=794).

Social Group	Remote Learning Proficiency 2020	Communication Challenges 2020	Connectivity Challenges 2020	Device Challenges 2020
All	2.6	3.4	2.4	2.1
Female	2.6	3.4	2.4	1.9
Male	2.7	3.5	2.5	2.3
Race/Ethnicity: Asian	2.7	3.5	2.4	2.0
Race/Ethnicity: Black	2.6	3.4	2.3	2.0
Race/Ethnicity: Hispanic	2.5	3.5	2.6	2.2
Race/Ethnicity: White	2.5	3.6	2.5	2.1
Multiple Races/Ethnicities	2.5	3.5	2.6	2.1
Economic insecurity	2.2	3.6	2.9	2.5
No economic insecurity	2.8	3.3	2.3	1.9
Financial hardship	2.3	3.5	2.8	2.4
No financial hardship	2.9	3.4	2.2	1.8
Need-based scholarship/loan	2.5	3.5	2.5	2.1
No need-based scholarship/loan	2.9	3.3	2.2	1.9
First generation student	2.4	3.6	2.6	2.3
Not a first generation student	2.7	3.4	2.3	2.0
In-state students	2.6	3.5	2.5	2.1
Out-of-state students	2.7	3.2	2.3	2.0
International students	3.2	2.9	2.3	2.2

**Table A2:** Weighed mean outcomes in 2022 (*N* = 1,106).

Social	Remote Learning Proficiency	Communication Challenges	Connectivity Challenges	Device
Group	2022	2022	2022	Challenges 2022
All	3	3.6	2.5	2.1
Female	3	3.6	2.5	2
Male	3	3.6	2.5	2.1
Race/Ethnicity: Asian	3.2	3.4	2.3	2.1
Race/Ethnicity: Black	2.8	3.7	2.5	2.1
Race/Ethnicity: Hispanic	3	3.5	2.6	2.1
Race/Ethnicity: White	3	3.7	2.6	2
Multiple Races/Ethnicities	2.9	3.6	2.7	2.1
Economic insecurity	2.6	3.6	2.9	2.5
No economic insecurity	3.2	3.7	2.3	1.8
Financial hardship	2.6	3.8	2.9	2.4
No financial hardship	3.5	3.5	2.1	1.7
Need-based scholarship/loan	2.9	3.7	2.6	2.2
No need-based scholarship/loan	3.4	3.5	2.3	1.8
First generation student	3	3.6	2.7	2.2
Not a first generation student	3.1	3.7	2.4	2
In-state students	3	3.7	2.5	2.1
Out-of-state students	3	3.7	2.5	2.1
International students	3.3	3	2.3	2.3

### Analytical design

Hypothesis testing was conducted using path analysis with a structural model constructed as shown on Fig 1. Weighted least square mean and variance adjusted (WLSMV) estimation was used due to the presence of binary endogenous variables in the model. Statistical tests were performed using the R platform version 4.2 and RStudio version 2023.03.1-446, along with the *lavaan* package version 0.6-15. To determine whether the hypotheses were supported, we examined the significance of individual paths and the global fit of the model to the observed data. A total of 4 cases had missing values for the model variables and were excluded from the analysis.

## Results

**Fig 3:**
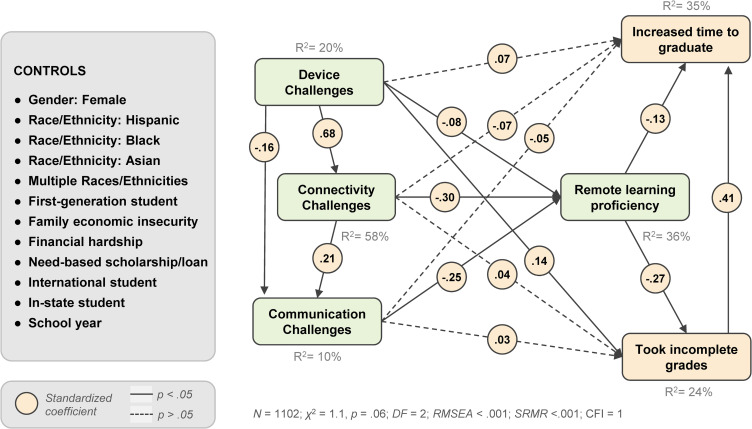
Structural equation model results, standardized coefficients.

The hypothesized model had an excellent fit (*N* = 1102; *χ*^2^ = 1.1, *p* =.06; *DF* = 2; *RMSEA* <.001; *SRMR* <.001; CFI = 1). The full model results are presented in Table 2 and Fig 3.

**Table 2:** Structural equation model results, standardized coefficients with standard errors included in parentheses.

Variable	Increased time to graduate	Took incomplete grades	Remote learning proficiency	Comm. challenges	Connect. challenges	Device challenges
Took incomplete grades	.47 (.04)***	-	-	-	-	-
Remote learning proficiency	-.13 (.04)**	-.27 (.04)***	-	-	-	-
Communication challenges	-.05 (.04)	.03 (.04)	-.25 (.02)***	-	-	-
Connectivity challenges	-.07 (.06)	.04 (.05)	-.30 (.04)***	.21 (.04)***	-	-
Device challenges	.07 (.05)	.14 (.05)**	-.08 (.04)*	-.16 (.04)***	.68 (.02)***	-
Gender: Female	-.13 (.04)***	-.03 (.04)	.01 (.02)	-.00 (.03)	.03 (.02)	.01 (.03)
Race/Ethnicity: Hispanic	-.02 (.05)	.05 (.05)	.01 (.03)	-.10 (.04)*	-.02 (.03)	-.01 (.03)
Race/Ethnicity: Black	.02 (.03)	.06 (.04).	-.03 (.03)	-.02 (.03)	-.01 (.02)	-.05 (.03).
Race/Ethnicity: Asian	-.05 (.04)	-.06 (.04)	-.01 (.03)	-.11 (.03)***	-.03 (.02)	.01 (.03)
Multiple Races/Ethnicities	.00 (.05)	-.04 (.05)	-.00 (.03)	.01 (.04)	.04 (.03)	.04 (.03)
Family economic insecurity	-.02 (.04)	.00 (.04)	-.01 (.03)	-.04 (.03)	-.04 (.02)*	.11 (.03)***
Financial hardship	.04 (.04)	.12 (.05)*	-.20 (.03)***	.17 (.03)***	.19 (.02)***	.34 (.03)***
Need-based scholarship/loan	.02 (.04)	.01 (.04)	-.05 (.03).	.00 (.03)	-.04 (.02)	.05 (.03)
First-generation student	.08 (.04)*	.03 (.04)	.02 (.03)	-.07 (.03)*	.01 (.02)	.04 (.03)
Working full time	-.03 (.04)	-.00 (.04)	.02 (.02)	-.04 (.03)	.01 (.02)	.05 (.02)*
International student	.09 (.05)*	.00 (.05)	-.02 (.04)	-.05 (.04)	-.08 (.03)**	.10 (.04)**
In-state student	.04 (.05)	-.09 (.05).	-.01 (.03)	.06 (.04)	-.03 (.03)	-.00 (.04)
School year (3-5)	.06 (.03).	.01 (.04)	.01 (.03)	-.03 (.03)	-	-
R^2^	35%	24%	36%	10%	58%	20%

. p <.1,

*p <.05,

**p <.01,

***p <.001.

The results supported H1, H2, and H3, which predicted that *remote learning proficiency* would be negatively associated with *device challenges* (*β* = -.08, *SE* =.04, *p* <.05), *connectivity challenges* (*β* = -.30, *SE* =.04, *p* <.001), and *communication challenges* (*β* = -.25, *SE* =.02, *p* <.001), respectively.

Supporting H4, *taking incomplete grades* was negatively predicted by *remote learning proficiency* (*β* = -.27, *SE* =.04, *p* <.001). In addition, incomplete grades were positively associated with *device challenges* (*β* =.14, *SE* =.05, *p* <.001) and *financial hardship* (*β* =.12, *SE* =.05, *p* <.05).

Supporting H5, *increased time to graduation* was negatively predicted by *remote learning proficiency* (*β* = -.13, *SE* =.04, *p* <.01), even after controlling for *taking incomplete grades* (*β* =.47, *SE* =.04, *p* <.001). After controlling for other model variables, *increased time to graduation* was also significantly positively associated with being a *first-generation* student (*β* =.08, *SE* =.04, *p* <.05), and an *international* student (*β* =.09, *SE* =.05, *p* <.05), and negatively predicted by being *female* (*β* = -.13, *SE* =.04, *p* <.001).

## Discussion

Researchers who study digital inequality frequently refer to three levels of inequality that are all important in their own right, but also, for how they compound each other in ways that explain how digital inequality actually functions within a population [32,33].

First-level digital inequalities are inequalities in *access* to the internet and digital devices. Our findings show that yes/no questions to capture whether or not a student has broadband internet or a laptop computer would not have been sufficient for capturing the inequalities in digital access experienced by under-connected students in the first two years of the pandemic [23]. Indeed, 95% of the Rutgers University-New Brunswick students that we surveyed in 2022 reported home broadband access—but, three in five of those students nonetheless reported at least one connectivity challenge, reflecting that their internet connectivity did not adequately support their remote learning needs at least some of the time. Likewise, 97% of surveyed students reported access to a laptop or desktop computer, but one in three of those students also reported at least one device challenge [23]. Therefore, fully accounting for first-level digital inequalities among college students requires accounting for digital access and for how well that access meets their learning needs.

Second-level digital inequalities broadly refer to whether individuals have the digital *capabilities* needed to achieve the goals they have for their digital engagement. Some researchers conceive of digital capabilities as digital skills that can be directly assessed; others rely on self-reports of digital confidence and competency, as we do here [34]. The specific digital skills that undergraduate students needed to be successful learners in remote instruction during the pandemic would have varied considerably by major and by the requirements of specific courses [21,31]. Our second-level measure of digital inequality had a different purpose: rather than measuring an objective set of skills, we were interested in students’ self-reported sense of competence that they could navigate remote learning environments successfully. We operationalized these measures as remote learning proficiency: students’ self-reports of their capabilities to understand instructors’ expectations, keep track of due dates and deadlines, and figure out new programs and platforms in remote learning environments. Hypotheses 1 and 2 were supported, showing that second-level digital inequalities (i.e., variations in remote learning proficiency) are tied to first-level inequalities in the forms of device and connectivity challenges (H1 and H2, respectively).

Remote learning proficiency is also negatively associated with students’ challenges communicating with their instructors (H3). When instruction was entirely remote, communicating with instructors necessitated digital devices and connectivity. And even in the hybrid course modalities that followed, mediated communication remained central to student-faculty communication. Furthermore, feeling supported and encouraged by instructors was essential for students’ developing remote learning proficiency because even in fully online learning environments, interpersonal connections between students and faculty remain essential for students’ success[2,35]. This finding is also consistent with extant research findings that developing digital competencies (i.e., the reduction of second-level digital inequalities) often relies on person-to-person trust and support, whether that support comes from a family member, a trusted friend, or an instructor [36,37].

Comparing the results presented here to previous research conducted at the same institution [2] offers additional insights into the trajectory of digital inequalities during the pandemic. As students and instructors got more used to online and hybrid classes, we found that remote learning proficiency increased from 2020 to 2022. At the same time, however, the device and connectivity challenges captured in our study did not decline much over time. We found no significant difference between the levels previously reported for 2020 [2] and those in 2022 examined here. This finding suggests that digital skills were improving with practice, but that first-level digital inequalities persisted.

Digital inequality scholars have amassed a considerable body of evidence tying first- and second-level digital inequalities together. van Deursen and Helsper [38] note the need to also document unequal *outcomes*—third-level digital inequalities—that concretely result from unequal digital access and skills. Efforts to account for third-level inequalities have been hampered, in part, by how difficult it is to define clear outcomes. Researchers can document, for example, if online searches were consequential for finding a new job, but outcomes that are not merely points in longer processes are hard to come by.

Timely completion of a college degree, and to a lesser extent, a college course, are an opportunity to document third-level outcomes of unequal digital access and unequal digital skills, as we have done with our analyses. We find that remote learning proficiency is negatively associated with taking incomplete course grades (H4) and with delaying time to graduation (H5), even after controlling for taking incomplete grades. First generation and international students were most likely to indicate having had to delay graduation, which suggests that college-educated parents may have been able to better buttress their children’s remote learning needs, and that remote learning across many time zones and international borders was especially challenging. We also find that delayed graduation was negatively associated with identifying as female, consistent with national findings about the disproportionate number of male students who struggled to persist in higher education through the pandemic [9].

The pandemic’s effects on undergraduate matriculation and persistence toward graduation threaten to undo a generation of progress in broadening access to higher education. Our literature review summarizes how historically marginalized students were disproportionately likely to experience cumulative stresses during remote and hybrid learning that made them more likely to stop out or drop out of college during the 2020-2022 school years. We also noted that students from lower-income backgrounds—students who are disproportionately first-generation and/or students of color—are also most likely to experience digital inequalities. Our analyses offer insights into how inequalities in digital access affected students’ confidence in their digital capabilities, and how those inequalities are associated with unequal outcomes in the short-term form of incomplete coursework, and the more worrying, long-term consequence of delayed college graduation.

Our findings should be especially alarming because they represent a “best-case scenario” sample of undergraduates who persisted in full-time student status throughout the first two years of the pandemic. Even so, we see the deleterious effects of being under-connected and of lower remote learning proficiency on on-time completion of individual courses and progress toward on-time graduation. Said differently, even among students who managed to persist full-time, digital inequalities produced materially unequal educational outcomes. Those inequalities will be starker still for the students who are *not* included in our sample: those who switched to part-time status or stopped out of college entirely because being under-connected was compounded by lacking childcare, work pressures, and/or changed financial circumstances [3,7,30].

### Study limitations

While our analyses provide important insights into the relationships between digital inequality and undergraduates’ learning experiences and outcomes, this study has some important limitations. First, and most importantly: although our data build on our 2020 study of undergraduates and our model confirms that the same associations exist between key variables in 2020 and 2022, both datasets are cross-sectional. We therefore cannot make the causal claims that longitudinal data would support, limiting the generalizability of these findings. Longitudinal data will be essential to fully understanding undergraduates’ challenges and experiences with digital inequality throughout the pandemic period, and the long-lasting effects of digital inequality during this period.

Second, there are ways that Rutgers University-New Brunswick may not be generalizable to other institutions of higher education. Rutgers is a public, four-year institution with an undergraduate population that is more diverse in terms of the proportion of students who are racial/ethnic minorities and first-generation students than the U.S. average, which makes it an especially rich study site for examining the unique effects of digital inequality. Rutgers also predominantly serves an in-state student population with a meaningful number of students who commute from home rather than living on or close to campus, and the state has less rural areas than would be the case at universities in other parts of the country.

Third, we note that we rely on students’ self-reports of their learning challenges and as to whether they took incomplete grades or have increased their time to graduation. The anonymous nature of the survey and the constraints of the Family Educational Rights and Privacy Act (FERPA), a federal U.S. law that protects students’ private information, prevented us from triangulating self-reports with the registrar’s records of how many incomplete grades were posted and how many students have filed delayed graduation dates. Registrar records would also be incomplete, however, because students may not have officially delayed their expected graduation date but know they will need to do so. We note that other studies of pandemic experiences have similarly relied on students’ self-reported changes to their graduation plans [39,40], with Aucejo and colleagues indicating that this approach “follows a small and growing literature that uses subjective expectations to understand decision-making under uncertainty” [27].

## Conclusions

The acute phase of the pandemic has had varied consequences for higher education. One is the need for digital inclusion protocols in every campus emergency preparedness plan. Epidemics, climate disasters, and other emergencies will necessitate rapid, short-term shifts to remote instruction for universities in affected regions. Post-COVID, the expectation will be that face-to-face classes can seamlessly move to remote learning during emergency campus closures since instructors are more comfortable and skilled with online teaching modalities and students are more familiar with them. But our findings show that the digital playing field is far from leveled post-pandemic [41]. Universities should routinely identify students who would be under-connected without their on-campus technology resources and have clear plans in place for sending those students home with a computer and/or for rapidly dispensing subsidies for home broadband access or prepaid WiFi hotspots to affected students.

The pandemic has also permanently altered routine college instruction. Whereas many instructors used online learning management systems peripherally for their courses prior to the pandemic, reports indicate these systems have remained more central to coursework in the return to in-person instruction [42]. As a result, under-connected students are likely facing increased challenges in their courses even in this post-emergency period. Our H4 results revealed that taking incomplete grades was positively associated with device challenges, while connectivity challenges had indirect associations. Students whose internet gets disconnected due to an unpaid bill or because they have hit the cap on their data plans can, if they must, log onto WiFi at a community or campus location, whereas students with underperforming devices have fewer creative workarounds for resolving those problems.

Our findings reveal that financial hardship was also positively associated with having to take incomplete course grades. In a qualitative study prior to the pandemic, Gonzales and colleagues [13] show that lower-income college students, who cannot afford to quickly repair or replace a malfunctioning device, often end up diverting time and energy from their coursework trying to address device challenges that their better-off counterparts would resolve within a day. Our findings suggest that those relationships between digital device quality and course performance persisted through the pandemic.

These convergent findings imply that even with a full return to campus, university leaders should be ensuring easily accessed on-campus device repair and students’ options for borrowing laptops for short- or long-term loan periods, in much the same way that campus libraries support students’ learning by loaning out books. Our results, summarized in Table 2, reinforce just how much students’ financial circumstances matter for their experiences of digital inequality even when controlling for racial/ethnic identities or first-generation status. The primacy of students’ financial circumstances in explaining who experiences inequalities in digital access, capabilities, and outcomes is an essential finding. The solutions for resolving under-connected students’ challenges with campus-provided resources are straightforwardly financial.

As researchers undertake the enormous task of accounting for the pandemic’s acute and ongoing effects on higher education, our findings underscore how fundamental a digital focus will be for explaining inequitable student experiences and outcomes—and for developing evidence-based initiatives to ensure more equitable student experiences going forward. It is imperative that college students not be “presumed connected” [17], either in the midst of crisis or more routine times, as digital tools and learning platforms become increasingly essential to higher education. Our hope is that our findings contribute to a fulsome discussion of how resolving digital inequalities can serve as an effective avenue for reducing more entrenched inequalities in relation to who is able to attend and graduate from college.
